# Recent Developments in Biomaterial-Based Hydrogel as the Delivery System for Repairing Endometrial Injury

**DOI:** 10.3389/fbioe.2022.894252

**Published:** 2022-06-20

**Authors:** Guiyang Cai, Zhipeng Hou, Wei Sun, Peng Li, Jinzhe Zhang, Liqun Yang, Jing Chen

**Affiliations:** ^1^ Department of Obstetrics and Gynecology, Shengjing Hospital of China Medical University, Shenyang, China; ^2^ Center for Molecular Science and Engineering, College of Science, Northeastern University, Shenyang, China; ^3^ NHC Key Laboratory of Reproductive Health and Medical Genetics (China Medical University), Liaoning Research Institute of Family Planning (The Reproductive Hospital of China Medical University), Shenyang, China; ^4^ Department of Cell Biology, Key Laboratory of Cell Biology, Ministry of Public Health, and Key Laboratory of Medical Cell Biology, Ministry of Education, China Medical University, Shenyang, China

**Keywords:** biomaterial-based hydrogel, endometrial injury repair, intrauterine adhesion, delivery system, IUA

## Abstract

Endometrial injury caused by intrauterine surgery often leads to pathophysiological changes in the intrauterine environment, resulting in infertility in women of childbearing age. However, clinical treatment strategies, especially for moderate to severe injuries, often fail to provide satisfactory therapeutic effects and pregnancy outcomes. With the development of reproductive medicine and materials engineering, researchers have developed bioactive hydrogel materials, which can be used as a physical anti-adhesion barrier alone or as functional delivery systems for intrauterine injury treatment by loading stem cells or various active substances. Studies have demonstrated that the biomaterial-based hydrogel delivery system can provide sufficient mechanical support and improve the intrauterine microenvironment, enhance the delivery efficiency of therapeutic agents, prolong intrauterine retention time, and perform efficiently targeted repair compared with ordinary drug therapy or stem cell therapy. It shows the promising application prospects of the hydrogel delivery system in reproductive medicine. Herein, we review the recent advances in endometrial repair methods, focusing on the current application status of biomaterial-based hydrogel delivery systems in intrauterine injury repair, including preparation principles, therapeutic efficacy, repair mechanisms, and current limitations and development perspectives.

## Introduction

The human endometrium is a dynamically remodeling tissue that lines the inner surface of the uterine cavity and is structurally divided into functional and basal layers (Salazar et al., 2017). As part of the menstrual cycle, the endometrium undergoes a monthly proliferative cycle (follicular phase regeneration), a differentiation cycle (luteal phase), and a shedding cycle (menstrual phase) induced by cyclic changes in estrogen and progesterone levels. (Salamonsen, 2003; Jabbour et al., 2006; Gargett et al., 2008; Mihm et al., 2011; Gargett and Ye, 2012). The proliferative changes in the follicular phase are affected by estradiol, and progesterone inhibits endometrial growth and plays a role in differentiation during the subsequent secretory phase. The onset of menstruation is due to the lack of trophoblast and human chorionic gonadotrophin (hCG) secretion in the late secretory phase, followed by an endometrial response of progesterone and estradiol withdrawal during luteal regression (Jabbour et al., 2006). The upper functional layer is where the physiological activities of proliferation, secretion, and degeneration occur and where the blastocyst implantation after conception (Strassmann, 1996). In contrast, regeneration usually occurs in the lower basal layer (Jabbour et al., 2006). In the normal non-pregnancy cycle, the endometrium area that does not fall off, fall off, and heals coexists, and the repair process is both rapid and scar-free. This periodic shedding ensures that the amount of exposed endometrium and damaged blood vessels at any given moment is minimized (Garry et al., 2009).

As the most common endometrial injury disease, intrauterine adhesions (IUA) refer to the various degrees of adhesion of the uterine cavity and cervical canal wall induced by various injury factors, and the endometrium is replaced by fibrotic tissue (Robinson et al., 2008). This condition was first described in detail by Asherman in 1948 and is therefore clinically known as “Asherman’s syndrome” (AS). Injury factors include repeated induced abortion, mid-term labor induction, uterine submucosal myomectomy and cesarean section, which can cause damage to the endometrial basal layer, endometrial repair obstacles, and fibrous tissue hyperplasia, resulting in the disappearance of the standard shape of the uterine cavity (Hooker et al., 2014). The clinical manifestations of IUA include abnormal menstrual bleeding or even amenorrhea, infertility due to uterine factors, recurrent miscarriage, placenta accreta, and other obstetric complications (Sun et al., 2018; Zhu et al., 2019). As one of the major causes of secondary infertility in women of childbearing age, IUA will hinder the implantation of blastocysts, damage the blood supply of the uterus and the early fetus, and eventually lead to miscarriage or complete infertility in patients, which has become an urgent public health problem for women of childbearing age.

According to different types of IUA, seeking active and effective treatment measures to separate adhesions, restore the normal shape of the uterine cavity, promote endometrial repair, and restore reproductive function is an urgent clinical problem to be solved, and it is also a hot spot and difficulty in clinical research in recent years. Traditional methods such as hysteroscopy adhesiolysis, artificial hormone therapy, and the placement of intrauterine devices are currently used clinically. Still, the therapeutic results obtained are minimal and remain poor effect in severe cases. There is insufficient evidence that these treatment modalities effectively promote regeneration or improve pregnancy outcomes after severe endometrial injury (Johary et al., 2014; Lin et al., 2015). In addition, the recurrence rate of severe IUA is as high as more than 62%, showing a poor prognosis despite an excellent initial treatment effect (Abudukeyoumu et al., 2020).

The introduction of various biological substances that stimulate tissue regeneration can promote endometrial injury repair (Cervelló et al., 2015; Almeida et al., 2021; Ma et al., 2021). Stem cell therapy is currently the most attractive therapeutic approach to repairing endometrial damage (Keyhanvar et al., 2021; Gharibeh et al., 2022). Among them, endometrial mesenchymal stem/progenitor cells (eMSC), a highly proliferative class of cells derived from the dynamic, cyclically regenerating human endometrium, show promise in regenerative medicine through paracrine immunomodulatory effects and enhancing endogenous stem cell function (Hennes et al., 2021). The pericyte and perivascular properties of eMSC indicate their specific roles in regulating angiogenesis, inflammation, and fibrosis (Thomas et al., 2017). Several reports have further demonstrated the feasibility of eMSC as a source of stem cells for regenerative medicine in uterine biology (Campo et al., 2017; Olalekan et al., 2017). The ease of sampling, culture potency and reduced rate of spontaneous differentiation into fibroblasts under specific culture conditions also serve as advantageous features in the application of eMSC (Gurung et al., 2015). Similar to eMSC, menstrual fluid-derived endometrial stem/progenitor cells (MenSC) have shown considerable potential in endometrial injury repair (Bozorgmehr et al., 2020; Wyatt et al., 2021). The results in the animal model showed a good repair effect (Zhang et al., 2016; Zhang et al., 2019). Then it was applied clinically to patients with severe AS, who showed varying degrees of increase in endometrial thickness and improved embryo implantation success, indicating that MenSC reduced fibrosis and promoted the repair of damaged endometrium (Tan et al., 2016). In addition, the endometrial stem/progenitor cell population includes the elusive endometrial epithelial stem/progenitor cells and a systematic review of the identification and use of epithelial stem cells in endometrial diseases (especially endometriosis) has been conducted (Cousins et al., 2021; Kong et al., 2021). However, stem cell transplantation for endometrial repair still has long-term safety and efficacy limitations, such as preservation difficulty, tumorigenicity, and low homing rates of transplanted cells. In addition, there is no uniform guidance or consensus available for a clinical reference regarding stem cell-related therapies (Bai et al., 2019; Song et al., 2021).

With the development of studies in recent years, some researchers have combined tissue engineering with regenerative medicine, using biomaterials for loading stem cells, therapeutic factors, or constructing *in situ* delivery systems as a therapeutic strategy for endometrial damage repair with remarkable results, thus opening up new avenues for the treatment of endometrium-related defects by integrating biomaterials with conventional therapies (Zhao et al., 2017). Biomaterials have the advantage of providing structural support that mimics natural endometrial tissue while allowing for the controlled release of drugs, growth factors, or other biologically active substances, with the expectation of more efficient drug delivery and improved therapeutic outcomes (Zhang et al., 2020a; Yoshimasa and Maruyama, 2021). Hydrogels have excellent water retention properties, swell in volume without dissolving and retain a certain elasticity, which is very similar to the properties of soft tissues in the body. The low interfacial tension and adhesion properties can reduce the irritation of the hydrogel to the tissue and provide good diffusion and longer retention time of the contents (Drury and Mooney, 2003; Peppas et al., 2006; Brandon et al., 2009; Milcovich et al., 2017). Therefore, hydrogels have become the most attractive carrier material for treating IUA or promoting endometrial regeneration. Herein, we focus on reviewing the different types of biopolymer-based hydrogels used to construct delivery systems such as estrogen, stem cells, or therapeutic factors for endometrial injury repair in recent years. The design principles and future trends of hydrogel materials are also discussed in anticipation of providing useful references for subsequent research.

### Design Principles of Hydrogel in Endometrial Repair

When hydrogels are used in the biomedical field, the design principles are often based on the specific microenvironment and application requirements of the target disease. In the treatment of IUA, the main objectives of hydrogel material applications are to prevent the occurrence of adhesions, repair endometrial damage and promote its regeneration. More specifically, the biocompatibility, biodegradability, mechanical properties, immunogenicity, ability to restore endometrial repair and reproductive function, controlled drug release properties, and even ethical issues of the candidate hydrogel matrix materials are all taken into account (López-Martínez et al., 2021a).

First of all, good biocompatibility is a necessary condition in biomedical applications. Most biomaterial-based hydrogels have excellent biocompatibility. Low immune rejection and biomimetics are the two most noteworthy indicators, as low immunogenicity ensures that the hydrogel and its loaded biomolecules are not removed as foreign bodies and can reach the injury to be effective. At the same time, biomimetics enhances the adhesion and growth of cells in the material and molecular responses, resulting in better repair. Secondly, the mechanical strength of the hydrogel needs to be sufficient to support the uterine cavity to reduce the formation of fibrosis, the degradation rate can match the repair process of the endometrium, and then it can be completely non-toxic metabolically excreted. Third, the hydrogel matrix can provide a gently controlled release of drugs, stem cells, or growth factors. Stem cell therapy is usually the first choice for treating severe IUA, but the delivery of hydrogels to stem cells is often insufficient. Ideally, a delivery system that promotes stem cell survival, proliferation, and directed differentiation can be constructed through hydrogels (Cervelló et al., 2015). In addition, the hydrogel formed *in situ* should have a suitable gelation time; too long time may cause the ungelatinized material to be diluted and washed by uterine fluid, unable to form a gel at the injury site, and too short a time may result in poor adhesion of the gel, which is not conducive to injury treatment (Lin et al., 2020). Finally, the sterilization steps of hydrogels. Generally speaking, the sterilization of the prepared hydrogel mainly goes through four stages: cleaning, disinfection, sterilization, and dehydrogenation. The most suitable sterilization method should be selected by comprehensively evaluating the hydrogel material itself, the preparation process, and the physicochemical properties of the loaded therapeutic agent.

### Application of Hydrogel in Endometrial Repair

Current research in the treatment of IUA is focused on the repair of the endometrium by using hydrogels as a matrix loaded with various bioactive substances. In the later sections, we will summarize the recent studies according to different sources of biomaterial-based hydrogel delivery systems (Thubert et al., 2015; Hooker et al., 2018) (Table 1).

**TABLE 1:** Summary of biomaterial-based hydrogels in repair of endometrial injury.

Active substance	Biomaterial	Biomaterial-based hydrogel features	Model	Mechanism	Effectiveness	References
Mesenchymal stem cell-secretome (MSC-Sec)	Cross-linked hyaluronic acid (HA) hydrogel	Biocompatibility, biodegradability, injectability, form-stability, low interfacial tension, adhesion properties and can improve the sustained-release effect of active substances	Murine uterus injury model	In research	Restoration of injured endometrial morphology and fertility	Liu et al. (2019)
The human placenta-derived mesenchymal stem cells (HP-MSCs)	Cross-linked hyaluronic acid (HA) hydrogel		Mice endometrium-injured model	Promoting the proliferation of human endometrial stromal cells by activating the JNK/Erk1/2-Stat3-VEGF pathway and the proliferation and migration of glandular cells through the Jak2-Stat5 and c-Fos-VEGF pathways	Reduced the fiber area, increased the endometrial thickness and the number of glands in the damaged endometrium, and improved embryo implantation rate	Lin et al. (2022)
Mesenchymal stem cell-derived apoptotic bodies (ABs)	Hyaluronic acid (HA) hydrogel		Murine endometrial acute damage model and rat IUA model	Decreasing the concentration of TNF-α & IL-1β and increasing the concentration of IL-10; the high expression of F4/80, CD163 and CD86; Increasing the number of Ki67 + cells and the increase in CD31 staining	Reduced fibrosis and promoted endometrial regeneration, resulting in fertility restoration	Xin et al. (2022)
Stromal cell-derived factor-1α (SDF-1α)	Chitosan-heparin hydrogel	Chitosan has good biocompatibility, biodegradability, and antibacterial activity, and heparin can enhance chitosan’s affinity with growth factors and promote its gelation	Uterine injury rats model	Long-term recruitment of hematopoietic stem cells (HSCs) that secrete additional VEGF and down-regulate TGF-β1 cytokine expression	Endometrial thickness, number of glands, and fibrosis level in the experimental group were not statistically different from normal uterus	Wenbo et al. (2020)
Growth factors (GFs)	Decellularized porcine endometrial extracellular matrix (EndoECM)	Biocompatibility, gelation at physiological temperatures, and slow resorption	Mice endometrial damage model	Increasing the secretion levels of PDGFbb, bFGF, and IGF-1	Increasing the number of endometrial glands, high cell proliferation index, new blood vessel development, and higher pregnancy rate	López-Martínez et al. (2021a)
Keratinocyte growth factor (KGF)	Heparin-modified poloxamer (HP)	Low toxicity and biocompatibility, good gelation properties after heparinization, excellent affinity with growth factors & Good bioadhesion, rapid gelation, enhanced mechanical properties and prolonged the retention time of KGF in the uterine cavity with the addition of EPL.	Rat IUA model	Ki67 and CD31 staining were increased, and the expression of LC3-II and P62 was elevated. The underlying mechanism is closely related to the activation of autophagy	The proliferation of endometrial epithelial cells and angiogenesis were promoted. The morphology and function of the damaged uterus were restored	Xu et al. (2017a)
Keratinocyte growth factor (KGF)	Heparin-modified poloxamer (HP) & ε-polylysine (EPL) as functional excipient		Rat intrauterine mechanical injury model	Inhibition of apoptosis in the damaged uterus	The proliferation of endometrial epithelial cells and glands was significantly enhanced, as was angiogenesis of the regenerating endometrium	Xu et al. (2017b)
17β-estradiol (E2)	Heparin-modified poloxamer (HP)		Rat IUA model	Improving the expression of kisspeptin via MAPKs p38 and ERK1/2 signal pathways & Activation of downstream signals PI3K/Akt and ERK1/2 inhibits endoplasmic reticulum stress signaling to play a protective role	Promoting endometrial proliferation and inhibiting apoptotic activity at the site of injury	(Zhang et al., 2020a), (Zhang et al., 2017)
β-estradiol (E2)	Aloe/Poloxamer	Bio-friendly, biomimetic and biodegradable properties, as well as being restorative, temperature sensitive, and low immunogenic	Rat IUA model	The levels of Ki67, cytokeratin, and estrogen receptor β were upregulated, while the expression of TGF-β1 and TNF-α was decreased	Promoting proliferation of endometrial mesenchymal cells, inhibiting their apoptosis, enhancing morphological recovery, and reducing the rate of uterine fibrosis	Yao et al. (2020)
Bone marrow stromal cells (BMSCs)	Pluronic F-127/Vitamin C	Hybridized hydrogels have lower toxicity and ensure a longer survival time of BMSCs at the injury site	Rat IUA model	The expression of cytokeratin, von Willebrand Factor (vWF), was restored, and the secretion of interleukin-1β (IL-1β) was inhibited at a low level	Thicker endometrium, more glands, less fibrotic areas, endometrium shows better recovery	Yang et al. (2017)
Adipose stem cell-derived exosomes (ADSC-exo)	Thiolated polyethylene glycol (SH-PEG)	Injectable, self-healing, degradable, antimicrobial, and microenvironmental protection properties	Rat endometrial damage model	Significant increases in VEGF, LIF, avβ3, and IGF-1 expression	Promoting neovascularization and tissue regeneration while inhibiting local tissue fibrosis and restoring fertility	Lin et al. (2021)
l-phenylalanine (L-Phe)	Poly (ethylene glycol)-b-poly (l-phenylalanine) block copolymer (PEBP)/PEG	Hydrophilic, excellent slow release, easily adjustable viscosity and mechanical strength	Rat endometrial damage model	Regulating the expression and interaction of TGF-β1 and Muc-4	Preventing fibrosis and promoting pregnancy in damaged uterine tissue	Wang et al. (2020a)
	Galactose modified xyloglucan (mXG)/hydroxybutyl chitosan (HBC)	Injectable, thermosensitive, cytocompatible, and hemocompatible	Rat repeated-injury model		Highly effective in preventing recurrent adhesions, promoting wound healing, and reducing scar formation	Zhang et al. (2018)
Decidualized endometrial stromal cells (dEMSCs)	Hyaluronic acid (HA)/fibrin	Biocompatibility, sufficient mechanical support, promoting cell growth and engraftment	Murine uterine infertility (synechiae) model	Expression and secretion of desmin, CD44, PECAM, and IGF-1	Reducing fibrous tissue and increasing endometrial thickness, restoring fertility	Kim et al. (2019)
Human induced pluripotent stem cell-derived mesenchymal stem cells (hiMSC)	Gelatin/sodium alginate	Cytocompatibility, porous structure, enhanced mechanical properties, and structural stability	Rat IUA model		Effectively prevent intrauterine adhesions	Ji et al. (2020)

### Natural Biomaterial-Based Hydrogels

Due to its unique biocompatibility and enzymatic biodegradability, Hyaluronic acid (HA) has been widely used in the field of biomedicine. Linear HA molecules were typically prepared using cross-linking techniques into hydrogels with a stable three-dimensional network architecture that provides the necessary structural and mechanical support for surrounding cells. In addition, the widely expressed hyaluronidase *in vivo* can completely degrade HA into non-toxic and harmless small molecular products (Weissmann, 1955; Koliakos et al., 2008). The cross-linked hyaluronic acid gel has been adopted and studied by many researchers as an excellent carrier for intrauterine administration.

**FIGURE 1:**
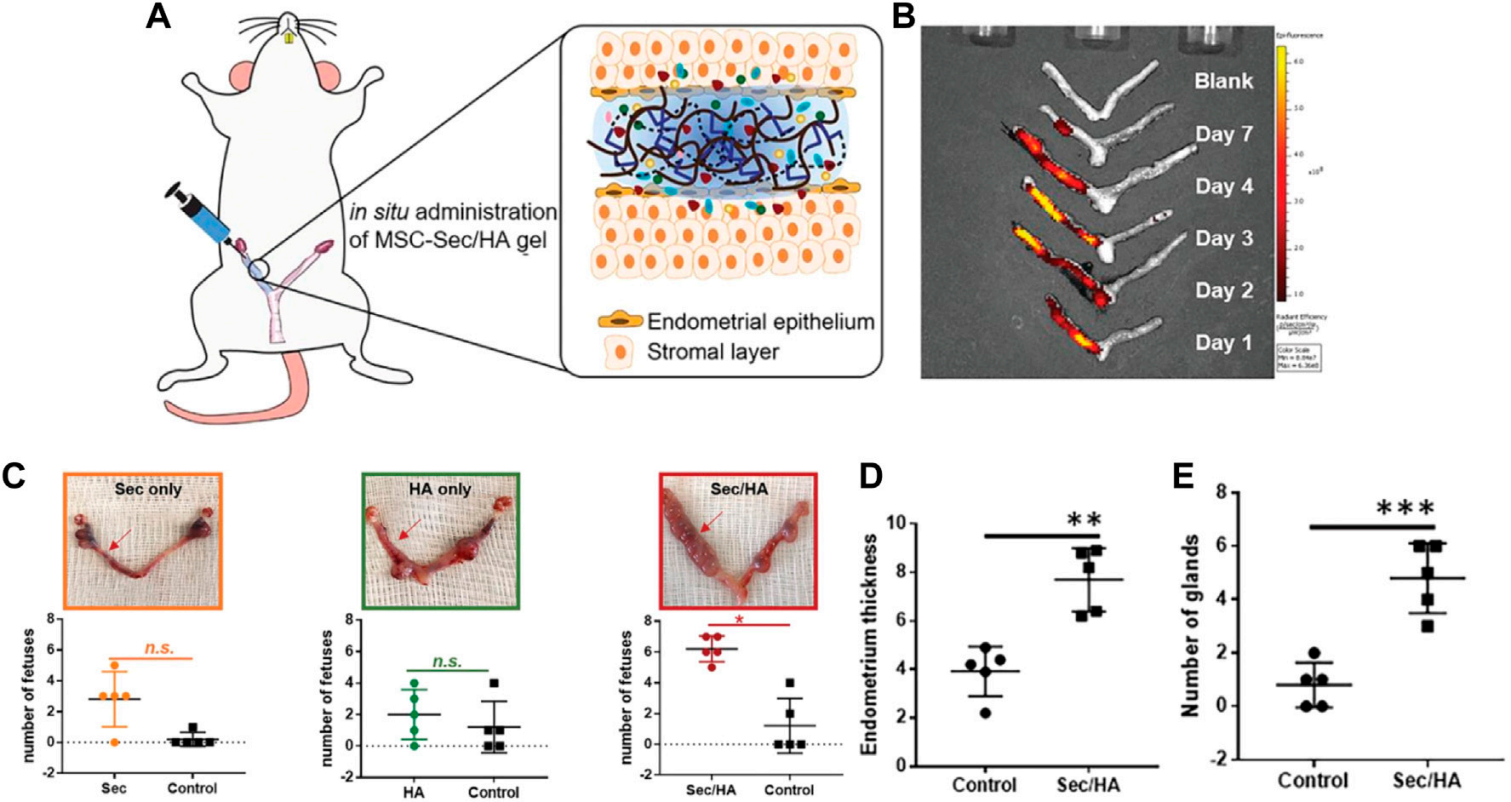
MSC-Sec/HA gel injection and a rodent model of endometrium injury **(A)** Schematic showing intrauterine injection of MSC-Sec/HA gel. **(B)** *Ex vivo* fluorescent imaging of rat uteri at days 1, 2, 3, 4, and 7 after injection. **(C)** Representative uterus images and quantitative data comparing the numbers of fetuses on both sides (red arrow indicates treated side). * indicates *p* < 0.05 when compared to the other side. **(D)** Comparison of endometrium thickness between the control side and the treated side of MSC-Sec/HA group. **(E)** Comparison of number of glands between the control side and the treated side of MSC-Sec/HA group. n = 5, ** indicates *p* < 0.01, *** indicates *p* < 0.001 when compared to the other group. Reproduced with permission from ref (Liu et al., 2019). Copyright 2019 WILEY-VCH Verlag GmbH & Co. KGaA, Weinheim.

Liu et al. reported a new strategy for constructing an intrauterine sustained-release system by combining the mesenchymal stem cell secretome (MSC-Sec) with cross-linked hyaluronic acid gel. MSC-Sec has a good effect on endometrial cells and endothelial cells. Then the labeled MSC-Sec were used to monitor the concentration and distribution of stem cells *in utero* by stereo fluorescence imaging technology to study HA hydrogel release kinetics. In the study, MSC-Sec-HA hydrogel was used to repair uterine damage in a rat model, and the research results showed that the endometrial morphology and fertility of the experimental rats were restored (Figure 1) (Liu et al., 2019).

**FIGURE 2:**
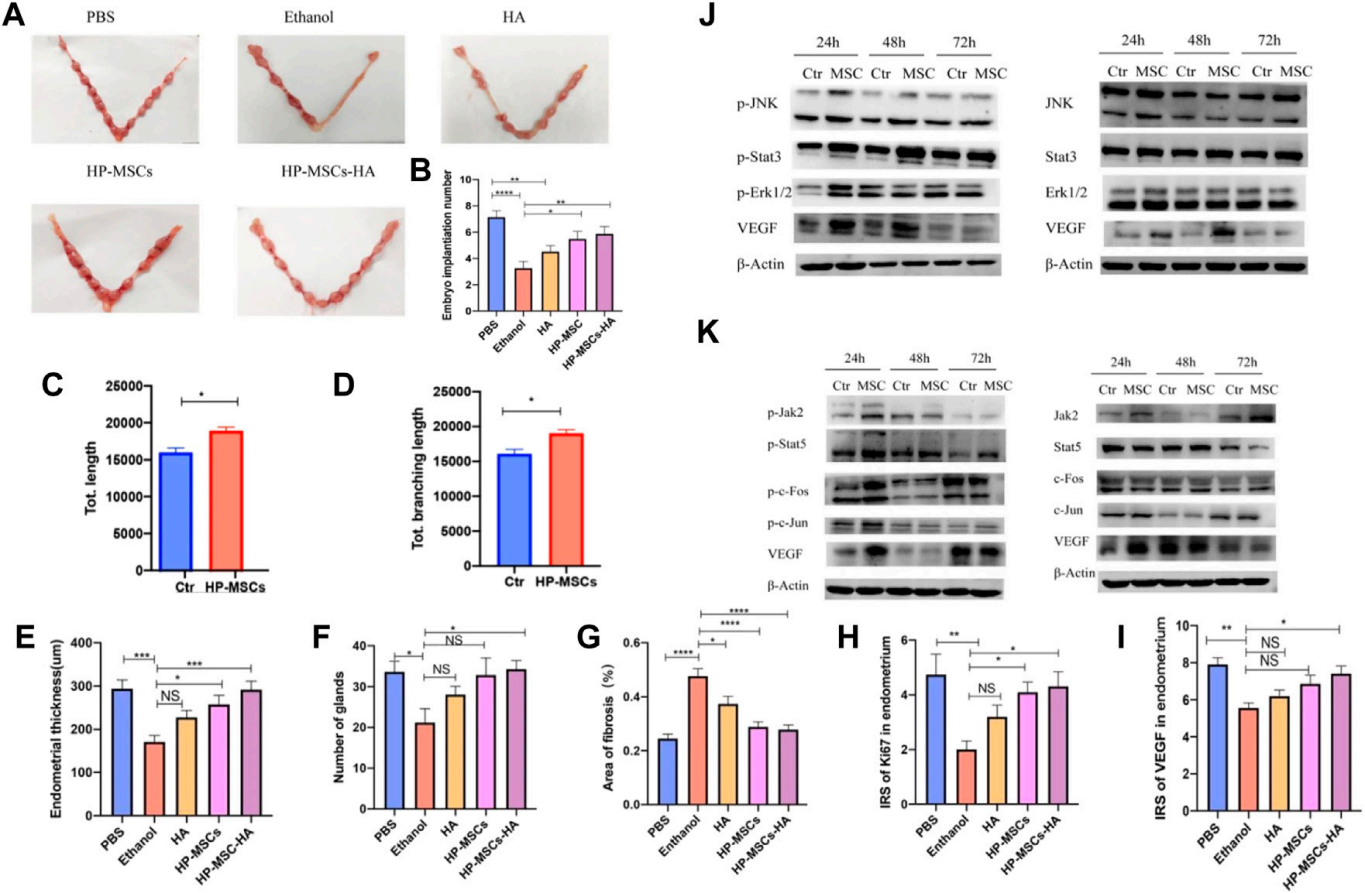
**(A,B)** Evaluate the endometrial receptivity of the five mouse groups with different treatments by the number of implanted embryos. **(C,D)** Quantitative assay of tube formation assay and data were expressed as mean ± SEM. * indicates *p* < 0.05. **(E)** Average endometrial thickness and statistical analysis (±SEM) of the five groups. * indicates *p* < 0.05, ** indicates *p* < 0.01, *** indicates *p* < 0.001, n = 6. **(F)** Average gland number and statistical analysis (±SEM) of the five groups. * indicates *p* < 0.05, n = 6. **(G)** Average fibrosis area and statistical analysis (±SEM) of the five groups. The ratio of the fibrotic area = endometrial fibrotic area/endometrial area. * indicates *p* < 0.05, ** indicates *p* < 0.01, *** indicates *p* < 0.001, **** indicates *p* < 0.0001, n = 6. **(H)** Statistic analysis of IRS of Ki67 in the endometrium of the five groups. * indicates *p* < 0.05, ** indicates *p* < 0.01, n = 6. **(I)** Statistic analysis of IRS of VEGF in the endometrium of the five groups. * indicates *p* < 0.05, ** indicates *p* < 0.01, n = 6. **(J)** p-JNK, p-Stat3, p-Erk1/2, VEGF, and corresponding total protein western blot analysis at 24 h, 48 h, and 72 h, respectively, after culturing without and with HP-MSCs, respectively. **(K)** p-Jak2, p-Stat5, p-c-Fos, p-c-Jun, VEGF, and corresponding total protein western blot analysis at 24 h, 48 h, and 72 h, after culturing without and with HP-MSCs, respectively. * indicates *p* < 0.05, ** indicates *p* < 0.01, **** indicates *p* < 0.0001, n = 8. Reproduced with permission from ref (Lin et al., 2022). CC BY 4.0. Copyright 2022 The Author(s).

The human placenta-derived mesenchymal stem cells (HP-MSCs) were loaded into cross-linked hyaluronic acid hydrogels to prepare HP-MSCs-HA hydrogels for the treatment of endometrial injury successfully. The collection process of HP-MSCs is safe and non-invasive (Zhu et al., 2013) and presents vigorous expansion ability and greater proliferation capacity (Feng et al., 2020). The low immunogenicity also ensures that no immune response will be caused during the treatment (Rinaldi et al., 1996; El-Toukhy et al., 2008; Cenksoy et al., 2013). Therefore, HP-MSCs are fully capable of becoming a new alternative source of stem cell therapy. Compared with the control group, the HP-MSCs-HA hydrogel system can significantly reduce the fiber area, increase the endometrial thickness and the number of glands in the damaged endometrium, and improve embryo implantation rate, demonstrating the good recovery of uterine function. The research group analyzed the potential treatment mechanisms. It concluded that the paracrine action of HP-MSCs could promote the proliferation of human endometrial stromal cells by activating the JNK/Erk1/2-Stat3-VEGF pathway and the proliferation and migration of glandular cells through the Jak2-Stat5 and c-Fos-VEGF pathways. This study provides a theoretical and experimental basis for the clinical application of HP-MSCs-HA in the treatment of endometrial injury (Figure 2) (Lin et al., 2022).

**FIGURE 3:**
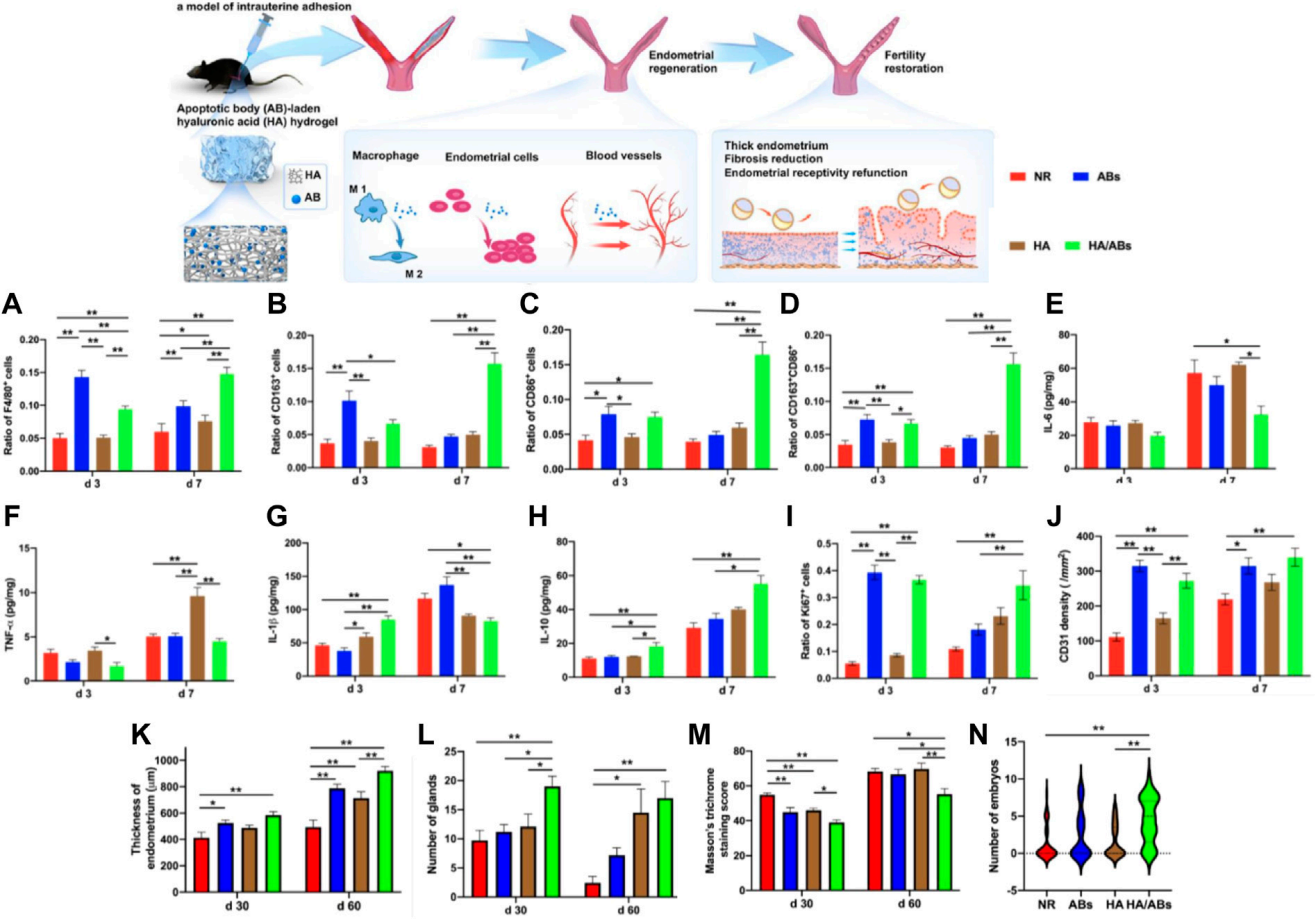
An apoptotic body **(A,B)**-laden hyaluronic acid (HA) hydrogel was designed to deliver ABs *in situ* to treat IUA in a rodent model. Statistical analysis was performed to examine the expressions of F4/80 **(A)** (marker of macrophages), CD163 **(B)** (marker of M2 macrophages), CD86 **(C)** (marker of M1 macrophages) and concurrent expression of CD163 and CD86 **(D)** (n = 8). ELISA for IL-6 **(E)**, TNF-α **(F)**, IL-1β **(G)** and IL-10 **(H)** in uteri under different treatments (n = 4). Statistical analysis was performed to examine the expressions of Ki67 (e) (marker associated with cell proliferation) and CD31 (f) (marker of capillary vessels) (n = 8). The results in a-d, **(I,J)** were normalized to the total number of cells per view under a ×400 magnification and expressed as a ratio of the total number of cells positive for a marker to the total number of cells present. **(K)** Quantification of endometrial thickness at days 30 and 60 post-surgery using H&E staining (n = 10). **(L)** Quantification of endometrial glands at days 30 and 60 post-surgery using H&E staining (n = 10). **(M)** Masson’s trichrome staining scores for uteri at days 30 and 60 after treatment (n = 10). **(N)** Violin plots showing the number of embryos per uterine horn following different treatments (n = 33 in the NR and ABs groups, n = 29 in the HA and HA/ABs groups). NR, natural repair without any treatment; ABs, injection of an AB solution only; HA, injection of HA only; HA/ABs, injection of the AB-laden HA hydrogel. *p < 0.05 and **p < 0.01. Reproduced with permission from ref (Xin et al., 2022). CC BY 4.0. Copyright 2021 The Author(s).

Xin et al. recently developed a cell-free therapeutic strategy for IUA treatment by combining mesenchymal stem cell-derived apoptotic bodies (Abs) with hyaluronic acid (HA) hydrogels to construct an Abs delivery system. Studies have shown that ABs contain a variety of functional biomolecules that play a crucial role in compensatory tissue regeneration and maintaining tissue homeostasis. They can induce macrophage immunomodulation, cell proliferation, and angiogenesis *in vitro* (Ryoo et al., 2004; Brock et al., 2019; Xu et al., 2019; Medina et al., 2020). The use of HA hydrogel delivery promoted the retention and sustained-release of Abs. In the mouse acute endometrial injury models and rat IUAs models, *in situ* injections of Abs-HA hydrogel significantly increased the thickness of the endometrium and the number of endometrial glands, effectively reducing fibrosis and promoting endometrial regeneration, thus restoring fertility. This study has validated the therapeutic potential of the Abs-loaded HA hydrogel, providing a clinically feasible cell-free therapy for endometrial regeneration and IUA therapy (Figure 3) (Xin et al., 2022).

A meta-analysis of recent clinical studies in which patients were administered the hyaluronic acid gel to prevent adhesions after intrauterine surgery was performed, with strict univariate and randomization principles followed in selecting clinical reports. The analysis results showed that the incidence of severe IUA in the HA gel group was significantly lower than that in the control group, while there was no significant effect on the incidence of mild adhesions. In addition, a broader clinical sample needs to be studied to determine the effectiveness and safety of HA gel in the fertility protection (Can et al., 2018; Liu et al., 2018; Fei et al., 2019; Fei et al., 2020; Mao et al., 2020; Zheng et al., 2020; Liu et al., 2022).

Qi et al. prepared a chitosan-heparin hydrogel through a mild process and verified the good stability of the material through *in vitro* release experiments. Subsequently, SDF-1α-loaded chitosan-heparin hydrogel was used to repair the intrauterine injury rat model. It was found by immunohistochemical staining and immunofluorescence staining that the treatment process was caused by hematopoietic stem cells recruited to the injury site, which promoted the recovery of the wound. And the recovery level of the experimental group was not statistically different from that of the control group. The experimental results demonstrate that the *in-situ* delivery of SDF-1α using chitosan-heparin hydrogel as a carrier can accurately repair the injured uterus in rats and be used as a candidate material for uterine injury healing and other wound dressing drug delivery systems (Wenbo et al., 2020).

Growth factors (GFs) therapy stimulates the body’s natural healing process by modulating the inflammatory response, promoting tissue formation, and inducing vascular regeneration (Farnebo et al., 2017). In recent years, researchers have often used biomaterials such as hydrogels loaded with GFs to prolong their retention time at the injury site to enhance the therapeutic effect. A bioengineered system based on decellularized porcine endometrial extracellular matrix (EndoECM) hydrogels (López-Martínez et al., 2021b) loaded with growth factors (GFs) was prepared. Proteomic analysis was performed to characterize the specific role of extracellular matrix proteins in tissue regeneration, followed by the repair effect of ECM hydrogels against endometrial injury in Asherman’s syndrome (AS)/endometrial atrophy (EA) after the addition of GFs was investigated. The animal experiments showed that the model mice treated with GFs-ECM hydrogel showed a series of regenerative effects with an increased number of endometrial glands, high cell proliferation index, new blood vessel development, and higher pregnancy rate. Therefore, this system is expected to be used for treatments related to endometrial repair in reproductive medicine (López-Martínez et al., 2021a).

### Synthetic Biomaterial-Based Hydrogels

Poloxamer is a class of water-soluble nonionic triblock copolymers with mild, non-toxic, and non-irritating properties approved by the FDA and included in the U.S. and European Pharmacopoeias. Its trade name is Pluronic. Poloxamer hydrogel is a synthetic thermosensitive hydrogel that undergoes a sol-gel transition at body temperature. Therefore, sol-state poloxamer has better fluidity, which is more convenient for developing and preparing injectable delivery systems. Studies have shown that poloxamer hydrogels are suitable for encapsulating cells or active factors, thereby promoting their release (Vashi et al., 2008). Likewise, adding heparin to the poloxamer hydrogel also plays a similar role to that mentioned above, enhancing the delivery system’s affinity for growth factors or hormones and prolonging the half-life *in vivo*. In addition, temperature-sensitive hydrogels have attracted the attention of researchers due to their unique properties. Temperature-responsive hydrogels used in biomedical applications are usually liquid below physiological temperature and can rapidly form gels at physiological temperatures around specific tissues. Most intriguingly, this type of hydrogel can be dynamically adjusted to the morphology and size of the uterus to create a bio-scaffold with excellent support after injection into the uterine cavity (Han et al., 2016).

**FIGURE 4:**
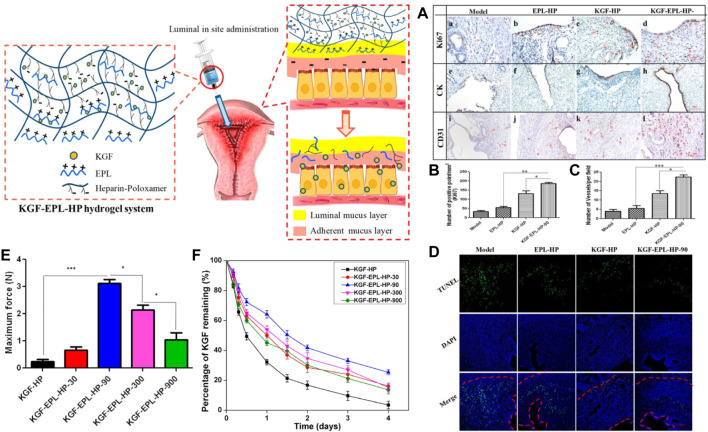
Scheme of thermos-sensitive bioadhesive KGF-EPL-HP hydrogel for injured uterus. **(A)** Immunohistochemistry images of Ki67 (a–d), CK (e–h), and CD31 (i–l) staining for injured uterus on day 3 after treatments. Staining-positive cells were marked by red arrows (scale bar = 100 μm). **(B)** Quantitative analysis of number of Ki67-positive cells, and **(C)** number of vessels per field at day 3 after surgery. Original magnification: ×200 (*: *p* < 0.05; **: *p* < 0.01; ***: *p* < 0.001 n = 3). **(D)** TUNEL assays kit analysis of the injury uterus on 3 days after treatment. Red line: the border of the basal layer; blue: cell nuclei, DAPI; green: apoptosis cells. Original magnification: ×200, scale = 1 μm. Adhesive evaluation of KGF-EPL-HP hydrogel. **(E)** The adhesive force of KGF-EPL-HP hydrogels against gelatin substrate in comparison with HP hydrogels. **(F)** The remaining percentage of KGF on excised rabbit uterine mucosa for KGF-EPL-HP hydrogels with various EPL concentrations after continuous rinsing with PBS (*p < 0.05; ***p < 0.001; n = 3). Reproduced with permission from ref (Xu et al., 2017a). Copyright 2017 American Chemical Society.

One study has prepared a temperature-sensitive hydrogel of poloxamer (HP) loaded with keratinocyte growth factor (KGF), modified with heparin to stabilize the growth factors and thus more easily control their release behavior. The rheological characterization indicates that the hydrogel system is suitable for intrauterine applications. Results from *in vivo* animal experiments showed that the HP hydrogel system significantly prolonged the residence time of KGF in the uterine cavity and promoted the proliferation and angiogenesis of endometrial epithelial cells (EEC), resulting in a better effect on the morphological and functional recovery of the injured uterus (Figure 4) (Xu et al., 2017a).

Subsequently, the team prepared mucoadhesive hydrogels loaded with KGF by adding ε-polylysine (EPL) as a functional excipient to a heparin-modified poloxamer matrix material to address two critical problems of short retention time and poor absorption of therapeutic agents or active factors in the damaged uterine cavity due to the rapid turnover of endometrial mucus. The rheological properties, adhesion, and KGF release behavior of HP hydrogels in the study were easily regulated by changing the doping amount of EPL in the formulation, which enabled KGF-EPL-HP hydrogels to promote the proliferation of endometrial epithelial cells and glands in a short time. As a result, the damaged endometrial morphology was well repaired, effectively solving the above two problems. By optimizing the matrix components, the research group made the prepared adhesive hydrogel system with great potential in endometrial repair (Xu et al., 2017b).

**FIGURE 5:**
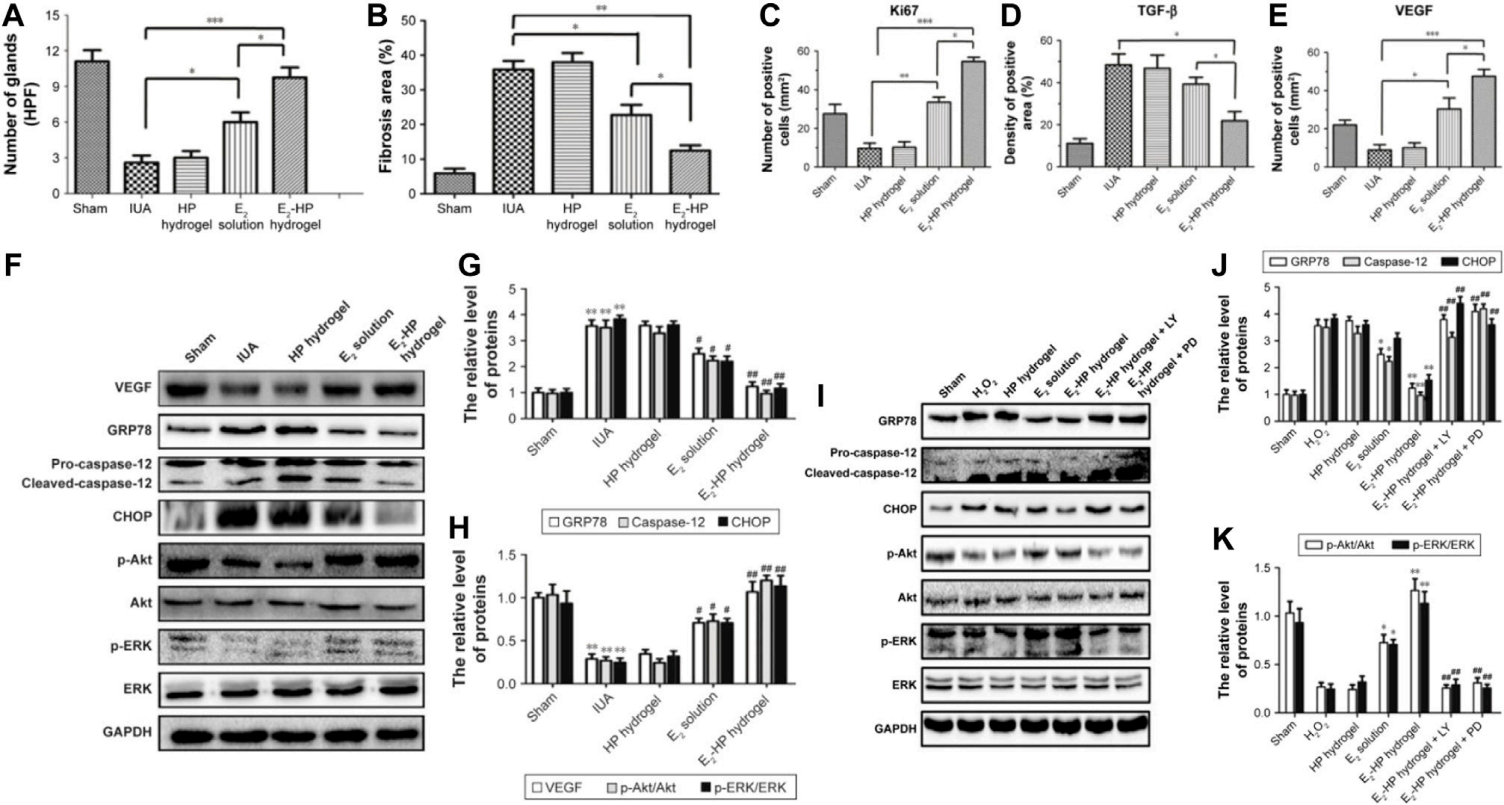
Schematic diagram of E_2_-HP hydrogel as an *in-situ* administration drug for the treatment of intrauterine adhesions. **(A)** Analysis of the number of glands in each group at 14 days after IUA. **(B)** Analysis of fibrosis area in endometrium in each group at 14 days after IUA. **(C)** Analysis of Ki67 positive cells of the immunohistochemistry results. **(D)** Analysis of TGF-β positive area of the immunohistochemistry results. **(E)** Analysis of VEGF-positive cells of the immunohistochemistry results. Data are presented as mean ± standard deviation; n = 5; *p < 0.05, **p < 0.01, and ***p < 0.001. E2-HP hydrogel inhibits ER stress and activates the Akt and ERK1/2 pathways in the IUA rats. Notes: **(F)** The protein expressions of VEGF, GRP78, caspase-12, CHOP, p-Akt, and p-ERK in each group were tested with Western blotting. GAPDH was used as the loading control and for band density normalization. **(G)** The optical density analysis of GRP78, caspase-12, and CHOP protein. **p < 0.01 versus the Sham group and #*p* < 0.05 and ##*p* < 0.01 versus the IUA group. **(H)** The optical density analysis of VEGF, p-Akt, and p-ERK protein. **p < 0.01 versus the Sham group and #*p* < 0.05 and ##*p* < 0.01 versus the IUA group. The activation of Akt and ERK1/2 is crucial for the protective effect of E2-HP hydrogel in H2O2-induced ER stress in EECs. Notes: **(I)** The protein expressions of GRP78, caspase-12, CHOP, p-Akt, and p-ERK1/2 in ER stress-induced apoptosis in EECs treated with E2-HP hydrogel and different inhibitors. GAPDH was used as the loading control and for band density normalization. **(J)** The optical density analysis of GRP78, caspase-12, and CHOP protein. **(K)** The optical density analysis of p-AKT and p-ERK protein. *p < 0.05 and **p < 0.01 versus the H2O2 group and ##*p* < 0.01 versus the E2-HP hydrogel group. Data are presented as mean ± standard deviation; n = 3. Reproduced with permission from ref (Zhang et al., 2017). CC BY 4.0. Copyright 2017 The Author(s).

In subsequent studies, the researchers prepared a novel estrogen sustained-release system by loading 17β-estradiol (E_2_) into heparin-modified poloxamer hydrogels. A rat injury model was established by simulating the scraping process. Then the prepared thermosensitive hydrogel was injected into the injured uterine cavity to investigate the repair effect of the endometrium. The experimental results showed that E_2_-HP hydrogel could effectively promote the proliferation of endometrium and significantly inhibit the apoptotic activity of the damaged area, which had a positive effect on endometrial regeneration. It was also demonstrated that E_2_-HP hydrogels are closely associated with inhibiting endoplasmic reticulum stress signaling through activation of downstream signals PI3K/Akt & ERK1/2 and the upregulation of kisspeptin through activation of ERK1/2 & MAPKs p38 pathways in IUA recovery. E_2_-HP hydrogel has emerged as a promising treatment for IUA (Figure 5) (Zhang et al., 2017; Zhang et al., 2020a).

As an ideal organic component, aloe was mixed with poloxamer to form a biofriendly hydrogel system in the study of Yao et al. (Baghersad et al., 2018), which is expected to be used to treat IUA with its ability that can promote wound healing (Kim et al., 2016; Singh et al., 2018). The researchers first prepared nanoparticulate decellularized uterus (uECMNPs) to encapsulate β-estradiol (E_2_) (E_2_@uECMNPs) to enhance the sustained-release effect of E_2_. E_2_@uECMNPs were then embedded into aloe/poloxamer hydrogels to make a nanocomposite temperature-sensitive hydrogel delivery system. The delivery system promoted the proliferation of endometrial stromal cells and reduced uterine fibrosis rates through stable and continuous delivery of E_2_ to the site of intrauterine damage in concert with multiple components. *In situ* imaging and *in vivo* histological analysis data further confirmed that the E_2_@uECMNPs-AP hydrogel exerted a good biological effect and exhibited excellent repairability, showing excellent application potential for the treatment of IUA (Yao et al., 2020).

Vitamin C can significantly affect the pluripotency, self-renewal, and differentiation of stem cells (D'Aniello et al., 2017). In Yang’s study, Pluronic F-127/Vitamin C hydrogels loaded with bone marrow stromal cells (BMSCs) were prepared. The results demonstrated that vitamin C significantly promoted the survival and proliferation of BMSCs in the system and attenuated the biotoxic effects of PF127 hydrogel. Thickening of the endometrium increased glands, and decreased fibrotic areas were observed after transplantation of the hydrogel into the uterine cavity of IUA model rats, which effectively promoted the endometrium regeneration. In summary, heparin, aloe, and vitamin C have all been used to functionalize poloxamer hydrogels to improve the therapeutic effect of IUA, and promising research progress has been made (Yang et al., 2017).

**FIGURE 6:**
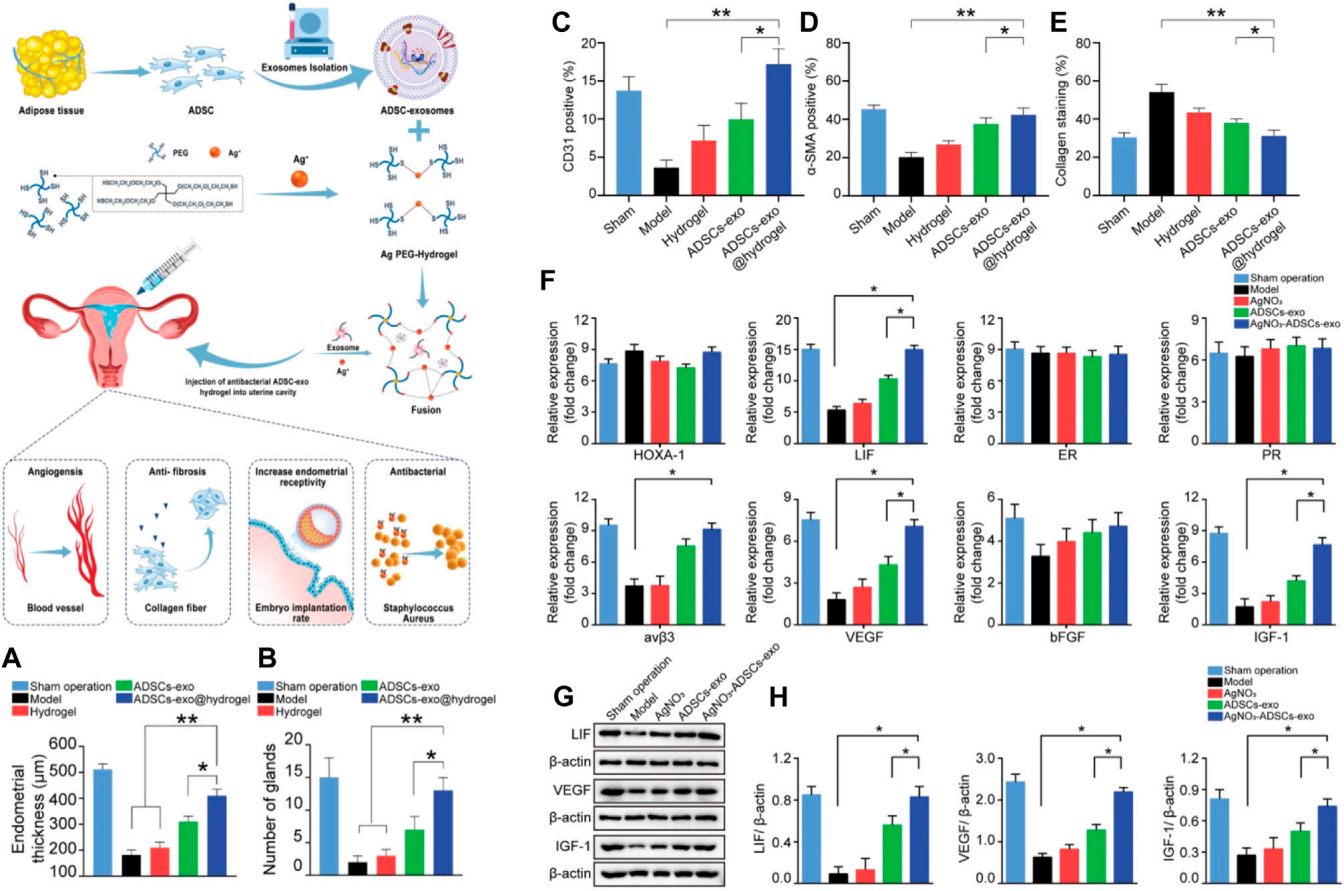
Schematic overview of the development of an ADSC-exo hydrogel for endometrial regeneration. **(A)** Endometrial thicknesses in each group. **(B)** Numbers of glands in each group. Administration of ADSC-exos or AgNO3+ADSCs-exo promotes endometrial neovascularization, myometrial regeneration, and reduced endometrial collagen deposition. **(C–E)** Quantification of CD31, α-SMA, and collagen protein expression levels. The impact of ADSCs-exo and hydrogel transplantation on the expression of markers of endometrial receptivity and angiogenesis. **(F)** A qRT-PCR approach was used to assess the expression of markers of endometrial receptivity (HOXA-1, LIF, ER, PR, Integrin β3, IGF-1) and angiogenesis (VEGF, bFGF), with β-actin serving as a normalization control. **(G)** Western blotting was used to assess LIF, VEGF, and IGF-1 protein expression in each treatment group. **(H)** Western blotting data of the levels of LIF, VEGF, and IGF-1 in different treatment groups. Data are shown relative to internal reference controls. Data are means ± standard error, n = 5. *p < 0.05, **p < 0.01. Reproduced with permission from ref (Lin et al., 2021). Copyright 2021 Wiley-VCH GmbH.

Both exosomes and apoptotic bodies mentioned above (Xin et al., 2022) belong to a subgroup of extracellular vesicles. Although they differ in diameter and mode of production, both have been used in tissue damage repair and have made some positive progress. The complex microenvironment of the endometrium often makes the treatment less effective than desired. Lin et al. developed an exosome injectable antibacterial PEG hydrogel for microenvironmental protection to address this problem. The exosome hydrogel was formulated by dynamic coordination of Ag^+^-S and fusion with adipose stem cell-derived exosomes (ADSC-exo). The system can promote the proliferation, migration, and tube formation of human umbilical vein endothelial cells (HUVEC) *in vitro*. And it can protect and improve the endometrial microenvironment *in vivo* for promoting angiogenesis and recovery of endometrial morphology and function while inhibiting local tissue fibrosis, thereby improving pregnancy outcomes. Although exosomes also have the disadvantage of not replicating *in vitro* compared to stem cells, nanoscale exosomes can easily pass through capillaries without the risk of immune rejection and tumorigenesis (Phinney and Pittenger, 2017; Zhang et al., 2020b). This study also provides a convenient, safe, and non-invasive approach for cell-free therapeutic strategies against IUA (Figure 6) (Lin et al., 2021).

**FIGURE 7:**
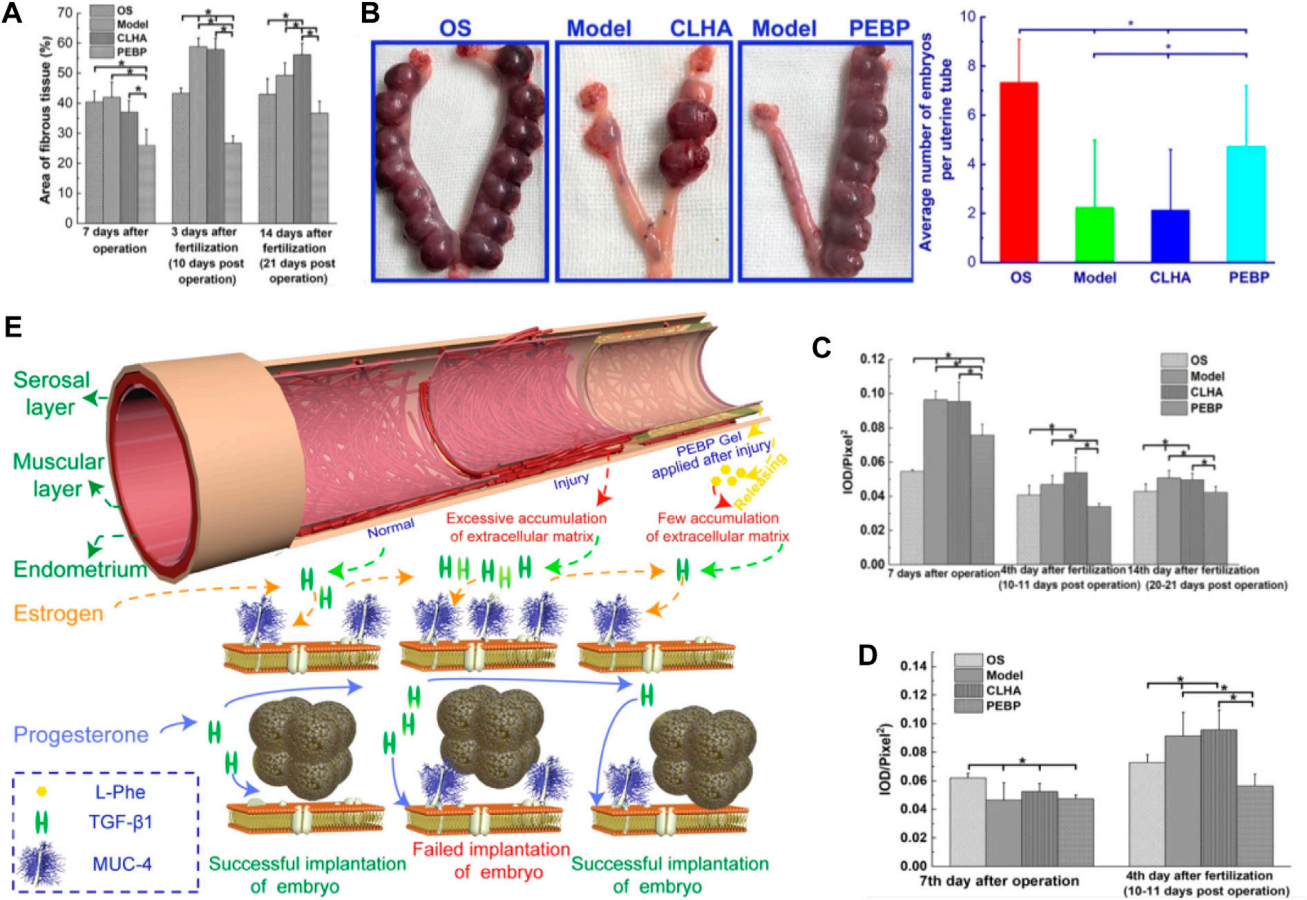
**(A)** average area ratio of fibrotic tissue with different treatments in the uterine stromal layer; and **(B)** average number of embryos implanted in uteri with different treatments on day 14 of pregnancy. **(C)** average IOD of immunohistochemical images of TGF-β1 expressed at the uterine stromal layer. **(D)** average IOD of immunohistochemical images of Muc-4expressed at the surface of the uterine wall. **(E)** Mechanism of PEBP/PEG hydrogel on embryo implantation after uterine curettage, by regulating interactions between TGF-β1 and Muc-4 expression. Reproduced with permission from ref (Wang et al., 2020a). Copyright 2020 American Chemical Society.

In order to prevent uterine fibrosis after injury, Wang et al. prepared a hydrogel material that can inhibit the proliferation of fibroblasts. Poly (ethylene glycol)-b-poly (l-phenylalanine) block copolymer (PEBP) was synthesized by ring-opening polymerization (ROP) of l-phenylalanine N-carboxylic anhydride initiated by methoxy polyethylene glycol amine. Subsequently, injectable PEBP/PEG hydrogels were formed through π-π accumulation between PEBP macromolecules and hydrogen bonding between PEBP, PEG, and H_2_O molecules. It was found that the PEBP/PEG hydrogel could sustainably release l-phenylalanine (L-Phe) through degradation, and L-Phe effectively inhibits uterine fibrosis and improves the success rate of embryo transfer by regulating the expression and interaction of transforming growth factor β1 (TGF-β1) and Muc-4. Therefore, this hydrogel material has potential applications in preventing uterine adhesions (Figure 7) (Wang et al., 2020a).

### Biocomposite Hydrogels

Natural hydrogels can usually interact with proteins or cells in specific or non-specific ways, enhancing the adhesion of the material in the uterine cavity. Thus, they affect the endometrial formation, cell growth, and phenotype maintenance. Still, their further application is often limited by disadvantages such as poor mechanical strength, uncontrolled degradation, risk of immunogenicity, and poor reproducible processing stability (Kirchhof et al., 2015; Rastogi and Kandasubramanian, 2019). Synthetic hydrogels can be prepared in large quantities, have more stable properties, and can be modified at the molecular level through polymerization, cross-linking, and functionalization. However, they cannot modulate intercellular responses, biorecognition, and cell-induced remodeling like native hydrogels. Therefore, in recent years, many researchers have combined the advantageous properties of various biomaterials to prepare composite hydrogels to obtain better repair effects (Lv et al., 2021).

Zhang et al. developed a thermo-responsive injectable hydrogel containing galactose modified xyloglucan (mXG) and hydroxybutyl chitosan (HBC) with good cytocompatibility and hemocompatibility. It has been shown the ability to promote skin wound healing and reduce scar formation in a rat trauma model. The synthesized hydrogel was found to be effective in preventing recurrent adhesions in a rat repeated injury-adhesion model. The conclusions of this study demonstrated the potential application of mXG/HBC composite hydrogel as an effective anti-adhesion system in the prevention and treatment of IUA-induced intrauterine adhesions (Zhang et al., 2018). In conclusion, several thermosensitive hydrogel delivery systems mentioned in the paper have made some progress in IUA treatment and expanded the ideas for developing stimuli-responsive hydrogels for endometrial repair in the future.

In another study, Kim et al. prepared a class of hyaluronic acid (HA)/fibrin composite hydrogels encapsulating decidualized endometrial stromal cells (dEMSCs), and a dose of thrombin was added to enhance gel formation and engraftment and can effectively release adhesion molecules. The results confirmed that the composite hydrogel had a good anti-fibrotic effect and increased the thickness of the endometrium in a short period of time. The transferred embryos were successfully implanted and developed normally, resulting in a live birth of the offspring. This study proposes an innovative therapeutic strategy with the efficacy of rapidly restoring endometrial damage and also has therapeutic potential in intractable infertility or recurrent miscarriage (Kim et al., 2019).

Ji’s research group used 3D printing technology to prepare a hydrogel scaffold loaded with human induced pluripotent stem cell-derived mesenchymal stem cells (hiMSC), and the 3D printing ink was made of a mixture of gelatin and sodium alginate. Compared to the direct local application of hiMSC, the hydrogel scaffold can significantly prolong the survival and action time of stem cells at the damaged site. *In vivo* animal experiments showed that the 3D printed hiMSC-loaded scaffold group promoted the recovery of endometrial tissue morphology and improved endometrial receptivity functional indicators. The damaged endometrium is well recovered (Ji et al., 2020). Feng et al. also developed a composite hydrogel containing different ratios of methacrylated gelatin (GelMA) and methacrylated collagen (ColMA) using 3D bioprinting technology and doped amniotic mesenchymal stem cells (AMSCs) in the hydrogel for the treatment of uterine adhesions. The hydrogels showed excellent anti-adhesive ability in *vivo* experiments and effectively prevented uterine adhesions in the IUA rat model (Feng et al., 2021). In addition, another research team used microfluidic droplet technology to prepare a new type of drug-loaded porous scaffold based on methacrylated gelatin and sodium alginate. The elasticity and mechanical properties of the scaffold have been improved, making it more suitable for the therapeutic repair of intrauterine injuries (Cai et al., 2019).

In addition, the methods for establishing animal endometrial injury models in the studies mentioned above are categorized and summarized. The first type is the mechanical injury method (scraping method), which usually involves repeated endometrial scraping through a surgical incision in the uterus after anesthesia until swelling and congestion to obtain an animal model of injury, and most studies have adopted this method (Xu et al., 2017a; Xu et al., 2017b; Yang et al., 2017; Zhang et al., 2017; Wang et al., 2020a; Zhang et al., 2020a; Wenbo et al., 2020; Yao et al., 2020; Xin et al., 2022). Some researchers have also established *in vivo* models of endometrial injury by intrauterine injection of ethanol (Kim et al., 2019; López-Martínez et al., 2021a; Lin et al., 2021; Lin et al., 2022). In addition, Liu’s group established a rat uterine injury model by using electrocoagulation, which is able to damage the endometrium and reduce angiogenesis, mimicking the pathological changes that lead to AS (Liu et al., 2019).

### Current Limitations

In the available reports, the mechanical strength of some hydrogel materials is so poor that they cannot provide sufficient mechanical support and even shift in position after implantation (Wang et al., 2021). And *in situ* biomaterial-based hydrogels with non-cytotoxic degradation byproducts, minimal fibrosis, and associated foreign body reactions are also critical to the application. Precise control of material degradation *in vivo* is required to guide *in situ* tissue repair or regeneration (Abdulghani and Mitchell, 2019). Furthermore, the hydration of the hydrogel can make the sterilization process of the formulation difficult and time-consuming (Lima et al., 2020). Current hydrogel-based delivery systems for the treatment of endometrial diseases also rely primarily on the degradation and diffusion of materials to deliver therapeutic agents (Liu et al., 2019). This release method lacks specificity and controllability, making it difficult to achieve the desired therapeutic effect. Although some researchers have developed temperature-responsive hydrogel delivery systems, as mentioned above, the role of the temperature-regulated release of therapeutic agents is still quite limited. Despite the fact that some achievements have been achieved in the research on the *in situ* delivery of estrogen and other therapeutic drugs by hydrogels, which effectively overcome the potential risks such as breast hyperplasia caused by oral estrogen, most of the available research data are still at the stage of animal experiments and clinical progress is limited (Wang et al., 2020b).

Similarly, the treatment of IUA with hydrogel materials combined with stem cell therapy is still in the research and exploration stage, and good results have been achieved in animal experiments. But the safety issues of stem cell therapy, such as tumorigenic complications caused by stem cell implantation, as well as low cell survival rate and high cost, have limited the further clinical translation of research results. The future research and development trend should be to prepare a multifunctional hydrogel delivery system for repairing endometrial damage, which can not only provide sufficient support for the formation of the uterine cavity but also can accurately and efficiently respond to various exogenous or endogenous trigger modes at the same time, it can carry a variety of therapeutic agents with the stable and sustained release (Lin et al., 2020; Wang et al., 2021).

It is worth mentioning that it is difficult to make practical recommendations for the clinical treatment of human endometrium-related diseases due to the diversity of animal models, the limited number of studies, and the uniqueness of the endometrium. Other than that, research on endometrial regeneration based on stem cells or growth factors is in its infancy, and the ethical and technical issues involved in the context of human studies make it a long way to go (Keyhanvar et al., 2021).

## Conclusion and Outlook

Hydrogel materials have received increasing attention in the field of endometrial repair due to their excellent water retention, biocompatibility, degradability, and controlled drug release properties, and early animal experiments have been performed to obtain more desirable therapeutic effects, showing great potential for clinical applications. Various types of hydrogels, including natural biomaterial-based, synthetic biomaterial-based, and biocomposite hydrogels, can be used as anti-adhesion physical barriers and can also be loaded with estrogen, stem cells, and various active factors (growth factors, exosomes) to build delivery systems for IUA treatment.

In conclusion, compared to cell delivery alone in stem cell therapy or a control group administering the active substance alone in an injury model, biomaterial-based hydrogels have many advantages as carrier materials for delivery systems: The hydrogels can provide sufficient dynamic mechanical support for the uterine cavity; their excellent adhesion and antimicrobial properties facilitate the regulation of the microenvironment at the site of endometrial injury and accelerate injury repair, and they have also been shown to enhance the sustained-release effect of cells or active substances and prolong the retention time in the uterine cavity. By promoting cell proliferation, differentiation, blood vessel formation, and regulating the intrauterine microenvironment to regulate the biological behavior of cells, it can more effectively enhance the repair of damaged endometrium, prevent the occurrence of adhesions, and improve the pregnancy rate.

The future development direction should focus on the role of hydrogels in repairing the endometrium and the exploration of therapeutic mechanisms. Under the premise of ensuring and optimizing safety, improving the bioactivity and biomimetic properties of hydrogels, and exploring the feasibility of the combined application of multiple therapeutic factors are crucial to accelerating the transition of the hydrogel delivery system from the experimental stage to the clinical translation. It can be foreseen that in the future, multi-disciplinary and multi-field resource integration and cross-cooperation will promote the vigorous development of hydrogel-based delivery systems in the field of repairing endometrial injury.
